# Integrating human‐centred design into the development of an intervention to improve the mental wellbeing of young women in the perinatal period: the Catalyst project

**DOI:** 10.1186/s12884-021-03675-y

**Published:** 2021-03-05

**Authors:** Tatiana Taylor Salisbury, Katie H Atmore, Inocencia Nhambongo, Muanacha Mintade, Luciana Massinga, Jak Spencer, Jonathan West, Flavio Mandlate

**Affiliations:** 1 Health Service and Population Research Department, Institute of Psychiatry, Psychology and Neuroscience, King’s College London, Denmark Hill, SE5 8AF London, UK; 2 Centro de Investigação em Saude de Manhiça (CISM), Manhiça Office, Rua 12, Cambeve, CP 1929 Manhiça, Mozambique; 3 Departamento de Saude Mental, Ministerio da Saude, Av Edurado Mondlane nr 1008, CP 264 Maputo, Mozambique; 4 Helen Hamlyn Centre for Design, Royal College of Art, SW11 4AN London, UK; 5 Faculdade de Medicina da Universidade Eduardo Mondlane, Avenida Salvador Allende nr 702 CP 257, Maputo, Mozambique

**Keywords:** Maternal mental health, Adolescent mothers, Intervention development, Public health

## Abstract

**Background:**

Mental wellbeing during pregnancy and the year after birth is critical to a range of maternal and infant outcomes. Many mental health interventions fail to incorporate stakeholder perspectives. The Catalyst Project aimed to work with key stakeholders in Mozambique to develop interventions and delivery strategies which were in-line with existing evidence and the needs, goals, and priorities of those both directly and indirectly involved in its success.

**Methods:**

A qualitative, human-centred design approach was utilised. Focus-group discussions, individual interviews, and observations with young women (aged 16–24 years), their families, community leaders, service providers and government were used to better understand the needs, priorities and challenges to mental wellbeing of young women. These findings were triangulated with the literature to determine priority challenges to be addressed by an intervention. Stakeholder workshops were held to identify potential solutions and co-develop an intervention and delivery strategy.

**Results:**

The 65 participants comprised 23 young pregnant women or new mothers, 12 family members, 19 service providers and 11 staff from the Ministry of Health. Participants highlighted significant uncertainty related to living situations, financial status, education, social support, and limited knowledge of what to expect of the impact of pregnancy and parenting. Family and community support were identified as an important need among this group. The Mama Felíz (Happy Mama) programme was developed with stakeholders as a course to strengthen pregnancy, childbirth and child development knowledge, and build positive relationships, problem-solving and parenting skills. In addition, family sessions address wider cultural and gender issues which impact adolescent maternal wellbeing.

**Conclusions:**

We have developed an intervention to reduce the risk of poor maternal mental health and gives young mothers hope and skills to make a better life for them and their children by packaging information about the risk and protective factors for maternal mental disorders in a way that appeals to them, their families and service providers. By using human-centred design to understand the needs and priorities of young mothers and the health and community systems in which they live, the resulting intervention and delivery strategy is one that stakeholders view as appropriate and acceptable.

## Background

### Importance of maternal mental wellbeing

Mental wellbeing, the ability to realise individual goals, cope with the stressors of life, enjoy work and be productive, and contribute to community [1], during the perinatal period (e.g. pregnancy and the year after childbirth) is critical due to its impact on physical and mental wellbeing and social outcomes of mothers and their children. Pregnant adolescents in low and middle-income countries (LMICs) often experience a variety of adverse events (e.g. family conflict, poor social support, poor self-esteem, gender discrimination, exclusion from education, poverty) which increase their risk for mental health problems [2]. An estimated 52 % of all Mozambican girls are married before the age of 18 [3] and 42 % of females give birth before the age of 18 – the 5th highest proportion globally [4]. While the scale of mental ill health among this particular group is uncertain, an estimated 30 % of deaths during pregnancy in the country are by suicide [5]. In the past decade, the government has taken significant steps to address the mental health of its citizens by approving the country’s first mental health policy and plan, increasing coverage to all of the nation’s districts through the training of non-mental health professionals to form a new cadre of health worker, the Psychiatric Technician [6].

### Efforts to improve wellbeing

Jewkes and colleagues [7] assert that addressing issues around teenage pregnancy must not only target prevention but also provide support during pregnancy and motherhood. Evidence-based promotion and prevention interventions to improve wellbeing among women during the perinatal period have been developed for use in LMICs [8]. However, fewer interventions specifically for adolescent and young women have been evaluated and only one in an LMIC (Chile; [9]). Wilson-Mitchell et al. [10] highlight the need for new and innovative models of mental health care for young mothers specifically designed for use in LMICs. Implementation of evidence-based interventions to improve wellbeing among young mothers is not just a health priority but a key component in the achievement of the United Nations’ Sustainable Development Goals [11], particularly, but not limited to Goals 1 (no poverty), 3 (good health and well-being), 8 (decent work and economic growth) and 10 (reduced inequalities). Unfortunately, the translation of ‘lab-tested’ mental health interventions to the real-world is often poor with limited input and ownership by the intended users and little or no flexibility in solving challenges as they arise. Successful implementation requires a deep understanding of the motivations, environment and conceptualisation of wellbeing and mental ill health in a given context, in order to increase the acceptability and uptake of such interventions. This is especially important for adolescents, as service providers are unlikely to share the same sub-culture, desires or experiences as their target group. However, the perspectives and experiences of young women and their families are largely missing from the literature [12].

The development of policies and interventions for early pregnancy and motherhood are largely detached from the realities of the young women they will impact. This is, in part, due to their lack of involvement in the process of development. The challenge in improving mental wellbeing is to engage young women, in the process of service delivery rather than simply providing what we think they need. Without their voices and the voices of those who act as their gatekeepers, any intervention will likely fail to be successful due to a lack of demand.

### Human‐centred design

Design, at its root, is more than art. It is undertaken by all people in their everyday lives to simplify activities and make them enjoyable. Human-centred design is an approach which actively engages stakeholders in the design process using cutting-edge methods to ensure interventions are optimised for both front-line use and local and national implementation. It involves five phases of intervention development:

1. Empathize – situation analysis and qualitative interviews with key stakeholders to identify perceptions, needs, goals and priorities;
2. Define – identification of the challenge to be addressed;
3. Ideate – development of potential interventions;
4. Prototype – refinement of interventions; and
5. Test – pilot evaluation and further refinement.

The UK Design Council [13] describe one aspect of good design as “a way to exceed user expectations, keep them happy, make them come back again and encourage them to recommend things to their friends” (p.29). Human-centred design allows not only for the active engagement of stakeholders in the identification and selection of wellbeing priorities and co-development of interventions but the inclusion of stakeholder-defined indicators of success which will be included in the evaluation of developed interventions [14]. The linking of interventions to indicators selected by stakeholders further increases their acceptability and scalability as stakeholders are able to see how the intervention fits into their needs and priorities.

Catalyst sought to marry the human-centred design approach with rigorous academic evaluation to develop an implementation strategy for existing effective interventions. It builds upon recent utilization of human-centred design within sexual and reproductive health in low resource settings [15]. This work demonstrates not only the feasibility of engaging adolescents in the implementation process but also the ability to use this approach in a context of high levels of associated stigma. Adopting non-traditional approaches to delivery of mental health interventions has the potential for the development of innovative services which may significantly improve the availability and uptake of services which improve mental health outcomes.

Catalyst aimed to partner with young mothers, their families and other key stakeholders to co-develop an intervention to promote mental wellbeing for young women during pregnancy and the year after birth. The objectives of the study were to:

1. understand the causes that affect the mental health of young Mozambican mothers during the perinatal period (i.e. pregnancy and the year after birth) through the experiences and priorities of young mothers in the District of Manhiça;
2. understand the experiences and priorities regarding mental wellbeing during the perinatal period among young women, their families, service providers and health system representatives (e.g. stakeholders);
3. identify a priority challenge for young women during the perinatal period that the research team and stakeholders intend to address; and.
4. co-design possible interventions to address the priority challenge selected by stakeholders.

## Methods

### Study Design

A qualitative design was used to develop and evaluate an intervention to improve mental wellbeing during the perinatal period among young women (aged 16–24 years). A human-centred approach was used to understand the conceptualisation of and priorities for wellbeing and mental ill health, identify a priority challenge(s) to wellbeing during the perinatal period, select and adapt existing mental health intervention(s) to address the challenge and develop a prototype for later evaluation. Over a four-month period, young pregnant women or new mothers, their families, and service providers were recruited from the Maragra Health Centre. Health system representatives from the Ministry of Health also took part. The study was approved by the Manhiça Health Research Centre Ethics Committee (CIBS-CISM/042/2018), Mozambique National Bioethics Committee (327/CNBS/19) and King’s College London Research Ethics Committee (HR-18/19-5877).

### Setting

**Fig. 1:**
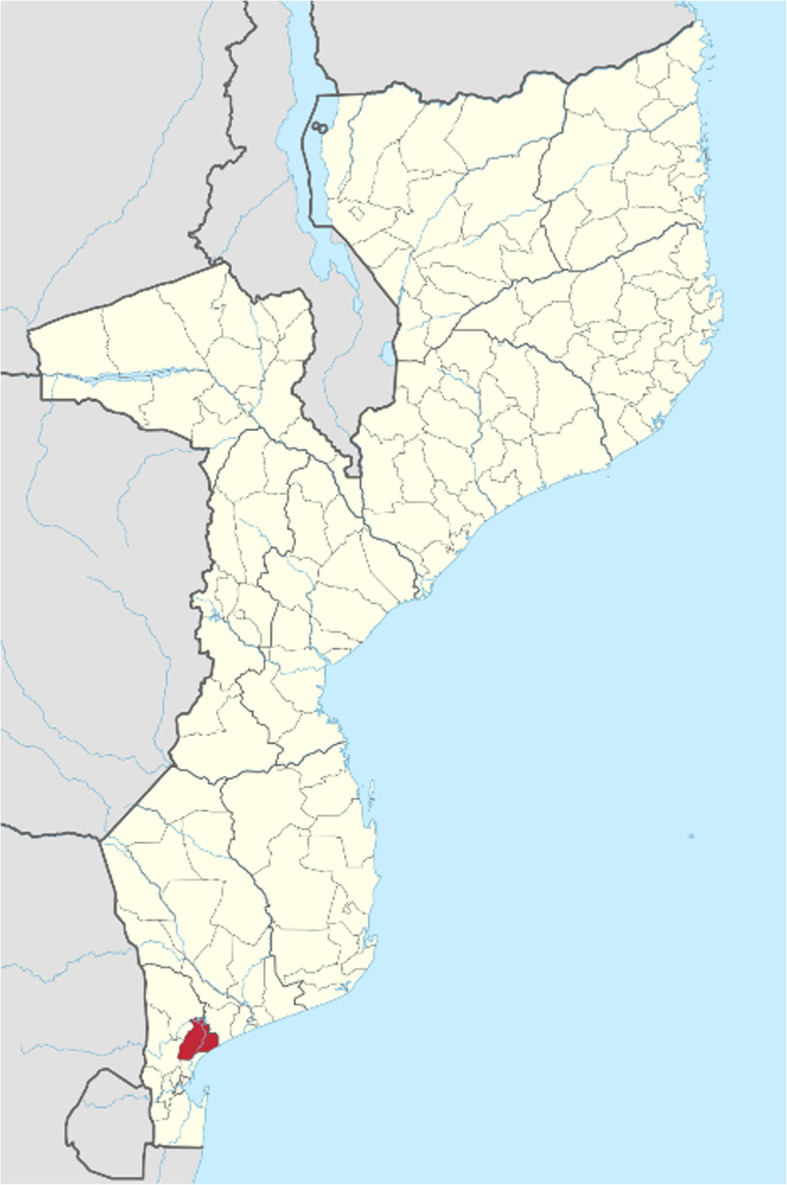
Location of Manhiça District. Source: NordNordWest, Manhiça District in Mozambique 2018

The study took place in the District of Manhiça, within the neighbourhood of Maragra and Maragra Health Centre. Manhiça District is located 80 km north of Maputo, the country’s capital (see Fig. 1). The district covers an area of 2,380 km^2^ and has a population of approximately 178,000. Manhiça District is served by 13 health centres and two larger hospitals (a district hospital and a rural hospital).

### Recruitment of participants/study population

**Fig. 2:**
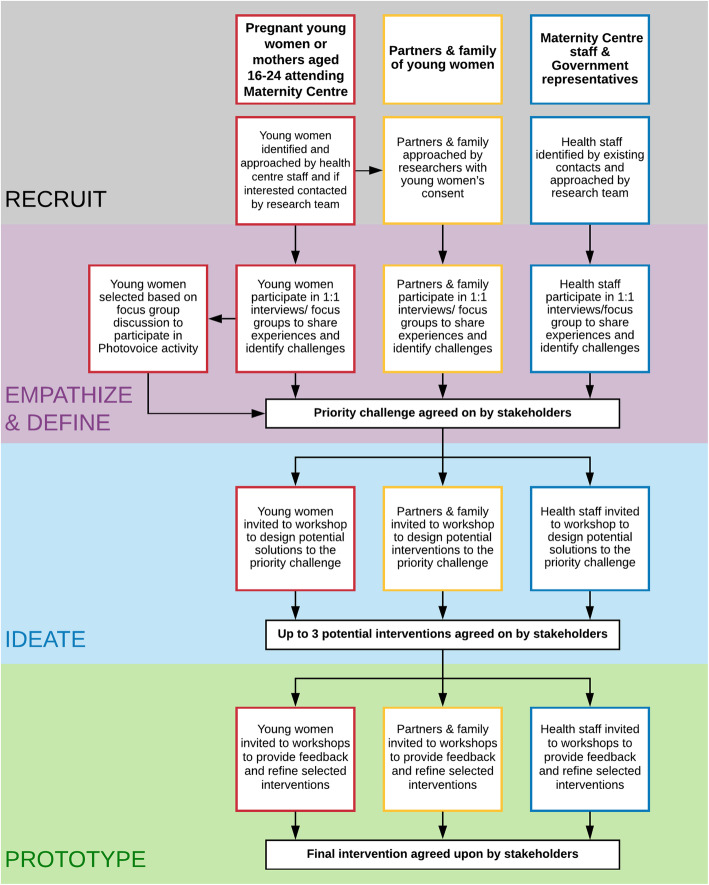
Study Flow Diagram

Young women (aged 16–24 years), their partners and family members, service providers, and Ministry of Health staff were recruited to develop the intervention. A description of sampling for the study is provided in Fig. 2.

#### Young women

Up to 20 young women (aged 16–24 years) who were pregnant or had given birth within the previous year and attended the Maragra Health Centre were eligible to participate. Young women were only be excluded from the study if they did not live in the district of Manhiça. Potential participants were identified by health centre staff to provide a sample representative of local socio-demographic characteristics including age, religion, income, marital status, education, and use of maternity services during pregnancy.Young women who met the study inclusion criteria were given information about the study by health centre staff and those interested were introduced to a member of the research team who provided the participant information sheet and consent form.Young women aged 16 and 17 years were allowed to participate without the consent of a legal guardian based on local regulations at the time of recruitment.

#### Partners and family members

Up to 12 partners and family members were recruited if they met the following inclusion criteria: (1) referred by a participating woman; (2) lived in the same household as the participating woman; and (3) aged 18 years or older.

#### Service providers and health system representatives

Up to 12 local service providers and health system representatives were invited to participate through existing contacts. Health centre staff were eligible to participate if they had worked at the health centre for at least 12 months.

### Data gathering procedures

#### Activity 1: empathize

One-to-one interviews and semi-structured focus group discussions (FGDs) were conducted with young women, partners or parents/in-laws, health and community service providers and policymakers based on participant preference and availability. Topic guides were tailored to each stakeholder group to elucidate their experiences, needs and priorities in relation to mental wellbeing during the perinatal period. All interviews were facilitated by at least one (for one-to-one interviews) or two (for FGDs) members of the research team and lasted up to one hour. Interviews and FGDs were conducted in Changana (the local language), Portuguese or English, based on participant preference. Summaries of each interview/FGD were drafted in English by members of the research team to guide subsequent interviews and workshops.

Up to 10 young women were invited to capture and document their experiences, needs, values and frustrations through the use of photography over a week using the photovoice method [16]. Consenting participants were given a digital camera and prompted to take pictures of situations which support or challenge their wellbeing. The women then participated in a FGD where they discussed their photos using Wang and Burris’s SHOWeD technique [17, 18].

A review of existing relevant maternal mental health and adolescent mental health promotion and prevention interventions was also conducted by the research team to identify potential interventions to inform the co-development process.

#### Activity 2: ideate

Workshops with young women and other key stakeholders were conducted to generate intervention ideas to the challenges identified in Activity 1. This inclusive process had two goals: (a) to identify existing evidence-based interventions as well as novel ideas from stakeholders and (b) to increase stakeholder feelings of ownership and the potential success of the final intervention.

#### Activity 3: prototype

Mock-ups (e.g. storyboards, role-play, diagrams, and videos) of selected interventions were initially created by the research team and shared with stakeholder to provide a visual representation of how the intervention would work. Up to three workshops were conducted with participants to refine the interventions and delivery methods through consensus.

## Results

Twenty-three young pregnant women or new mothers, 12 partners or family members, and 19 service providers were recruited from the Maragra Health Centre. Eleven health system representatives from the Ministry of Health also took part. Most commonly, participants were just under 20 years of age (range: 16–24 years), currently pregnant, living with their partners, and not attending school or working.Partners and family member participants were comprised of partners, mothers, fathers, mothers-in-law, and cousins. Service providers were all based at the Maragra Health Centre and had been in their roles for an average of 8.2 years (range: 5 months – 35 years). All policy makers were based in the Mental Health Department at the Ministry of Health.

### Experience of young women during the perinatal period

The majority (61 %) of young mothers highlighted uncertainty over their futures as a significant challenge they face. Their uncertainty focused on four domains: pregnancy outcomes, parenting, education, and financial stability. Participants did not feel that they understood the changes to their body and the development of their babies during pregnancy and how to take care of themselves physically and emotionally during this time.

*Only in the last few months I have been afraid… afraid of what was going to happen, how the childbirth was going to be.* Young woman, 3 months pregnant.

This was also emphasised by family and service providers as an issue for young mothers.

*I saw a 15-year-old teenage mother, she has a baby who must be a year old. I diagnosed low weight. The mother did not know how many times she had to feed the baby. The baby ate during the day, and cried until dawn at night, the baby came here all skinny, she didn’t know that she has to wake up to give breast milk.* General medical technician.

Although several (39 %) were happy about their pregnancy and felt emotionally and materially supported, a sub-set of young women, particularly those with unplanned pregnancies, had mixed or negative feelings towards the pregnancy and the impact it would have on their lives and that of their children.

*Well, I was not excited because the pregnancy was not planned. It was something that happened….* Young woman, 3 months pregnant.

*[I] think about how I’m going to give birth. If everything it’s going to be okay or not.* Young woman, 9 months pregnant.

*How am I going to take care of the baby if I’m not working.* Young woman, 5 months pregnant.

There is significant tension between continuing education and finding employment to support the household. A third of young women remained in school during and/or after pregnancy. This included mothers across the age range (e.g. 16–24 years). Six participants identified their pregnancy as the reason they left school. This was due to increased travel distances after moving in with their partner, fatigue, and perceived stigma associated with pregnancy at a young age. Five young mothers expressed a wish to return to school following their pregnancy. However, those in school and those who wished to return identified the need to work, lack of time, lack of childcare, and school fees as challenges to returning to school.

*My concern is after I give birth, I don’t know who the baby will be with when I go to school.* Young woman, 9 months pregnant.

*I don’t know, if the second child accepts formula I will go back to school. …I stopped studying because this one didn’t accept formula… I couldn’t leave him [at home] and go to school.* Young woman, 4 months pregnant.

Four family members of young women highlighted the need for them to remain in or return to school. These families viewed education as the path to financial independence and part of their responsibility in supporting her through pregnancy and motherhood. A young women’s decision to leave school may be viewed as a failure by her family and create a barrier to support. In all four families, the young women did not leave their homes to move in with her partner.

*I have been advising my daughter that despite what happened, when the child grows up she has to go back to school because by going to school you have the independence to do what you want in life. If she doesn’t find a job I could help her to enter the police academy so that she becomes a policewomen.* Father.

*My cousin is an orphan. She became pregnant at the age of 18 (…) the man who got her pregnant abandoned her for fear of taking responsibility because men now use girls and don’t take them seriously. We told her not to stop going to school. She continued until she gave birth, she had a rest and went back to school until now we support her, we stay with the child while she goes to school.* Cousin.

Where young women live with their partners and/or their families, financial contribution and/or taking on household responsibilities may be more important than completing education. Financial stability and their contribution to household finances was important to many young women.

*Her [daughter-in-law’s] plans, were to finish school and take a professional course, but since I am a seamstress, I taught her how to sew and what she would like is to have her own sewing machine and then she could go on with her life.* Mother-in-law.

Sixty-five per cent (*n* = 15) of young mothers in our study had left their family home and were currently living with their partner and/or his family. Thirty per cent (n = 7) of young women identified employment as a current goal.

*My dream is to work in the beauty salon.* Young woman, 9 months pregnant.

Although there was strong agreement among partners, family members, service providers, and Ministry of Health officials that partners and families must support young women during pregnancy and the year after birth, this was not always the case. Instances of fathers and their families failing to accept paternity and provide support, and young women’s own families pushing them to leave home are not uncommon.

*We as her parents continue to support her just as we support her baby because we don’t even know his father, the whole burden of expenses is ours.* Father.

*Ohhhh… I have no one to help me, I help myself, I look for a job and work but nobody helps me.* Young woman, 7 months pregnant.

Incompatibility of available services to support pregnant young women were described by service providers. Sexual and reproductive health is provided to adolescents through specialist young people’s services (known as SAAJ) which are located in separate offices within the district hospital and at secondary schools.However, once a young person becomes pregnant her responsibility for her care is assumed by the maternity team.

*Well, we may be able to lead these cases through lectures in schools, talking more about family planning, pregnancies (…) and they should also have a kind of lectures that is for them to know how to behave after childbirth, and try to do different for the welfare of the baby as well as hers because some mothers go into depression after childbirth.* General medical technician.

Negative attitudes towards early pregnancy were sometimes identified within the health clinic. Young women were described by some health providers as difficult to work with and irresponsible for not following guidance given by health providers.

*Ehhh it is difficult to explain the diagnosis, explain the importance of following the treatment because they are also children, it is difficult to give treatment to a child. They even say grandma will do it, but grandma is not here, so how will she explain it to grandma later? So it is difficult to certify that this baby will be given the correct medication, at the right time and improve.* General medical technician.

It is in these settings young women feel vulnerable to the negative attitudes associated with early pregnancy.

*I delivered myself…the nurses were asleep…I just had courage at that moment…they just cut it off (the umbilical cord) (…) then they took the baby and did other things.* Young woman, new mother.

The key challenges for young women during the perinatal period agreed by stakeholders were:

- Poor understanding of how to take care of their physical and emotional health;
- Poor understanding of how to take care of a baby;
- Changes in life circumstances (e.g. moving home, leaving school, new household responsibilities);
- Need for financial stability; and
- Need for social support.

### Existing maternal mental health prevention interventions

Within the literature, 14 studies of interventions to improve the mental health outcomes of adolescent and young mothers were identified. The majority (93 %) of interventions were conducted in North America with one study carried out in Chile. Forty-three per cent (*n* = 6) of studies were evaluated in a sample of less than 100 participants. The interventions were largely targeted at-risk groups identified by high prevalence of teen pregnancy and/or poor mother and child outcomes. These interventions had several common components: communication skills, family planning, parenting skills, goal identification, coping skills, relationship skills, linkage to health and community services. Common elements included developing a strong therapeutic alliance, a strengths based-approach, individualisation and social support. Interventions were mostly in-home, individual sessions provided by trained health workers. Evidence of impact on mental health outcomes were mixed.

### Intervention development

During ideation sessions, potential solutions to address the challenges were discussed. Educational sessions to teach young mothers basic parenting and self-care skills was identified as a core component for an intervention. Participants felt the intervention should also be tailored to focus on individual goals and providing practical support and advice to develop a pathway to reach them. Extension of the intervention to include education of partners and family members to reinforce support was also suggested. Service providers highlighted the need to strengthen the identification of young mothers who were experiencing mental health difficulties and their referral to appropriate services. A storyboard depicting a young women’s journey through the intervention was developed by the research team based on stakeholder discussion and shared with stakeholder groups for refinement in two iterations.

### The Mama Felíz programme

The Mama Felíz (Happy Mamma) programme is a motherhood preparation course which begins shortly after a young woman attends her first antenatal appointment. Participants are identified by the maternity nurse (over 90 % of mothers in the community attend antenatal care) and referred to a member of the Mama Felíz team to schedule attendance at a group information session at the health clinic.Participants are grouped into classes by delivery date and home address to strengthen their local support network. Sessions focus on understanding changes experienced during pregnancy and how to take care of their bodies and feelings during this time, building positive relationships, problem-solving, childbirth, child development, and parenting skills. In addition to group sessions, one-to-one sessions in the woman’s home or health facility are held to better understand each woman’s goals, screen for mental health problems, and to provide family educational sessions to partners and other key members of her household. These sessions will focus on the importance of mental wellbeing during this time, a discussion of their views on the matter and training in the ways in which they can better help the young woman, her baby and each other in creating a supportive and nurturing home environment. Our strengths-based intervention will be delivered by extensively trained mothers in the community and will incorporate components from existing evidence-based interventions.

## Discussion

While much of the global effort has focused on reducing pregnancies, less energy has been focused on supporting young mothers through pregnancy and motherhood. Early pregnancy and motherhood results in significant changes in a young woman’s life. While the perinatal period increases vulnerability for negative outcomes for young women and their infants, pregnancy and motherhood can also result in increased agency to improve one’s life [19, 20]. To date, few perinatal mental health interventions specifically for young mothers exist. The Catalyst project aimed to work with young women, their families, service providers, and government representatives to co-design and intervention to support young women’s mental health. Five challenges to the wellbeing of young mothers were identified as stakeholder priorities: uncertain future; financial stability; physical and emotional health; parenting; and social support.

All stakeholders highlighted the challenge of an uncertain future during pregnancy and early motherhood on young women’s mental health. The recent government ban on child marriage [21] will likely reduce the numbers of teenagers marrying as a result of early pregnancy. However, there may still be uncertainties over with whom she will live. There is also uncertainty surrounding her educational future. Despite efforts to improve education within the country [22], two-thirds of young women interviewed were not in education. One-third of those not in school identified their pregnancy as the reason for not attending. This is consistent with existing evidence in Mozambique [22, 23]. In 2003, legislation was introduced which requires girls who become pregnant to leave their schools and transfer to night courses to complete their education, as opposed to being excluded. While providing pregnant youth with a pathway to academic achievement, the law places additional obstacles (e.g. transport, vulnerability at night, poor quality instruction and childcare) which prevent many from attending [24].

Among young women who live with their partner and his family, there was a tension between education and financial stability of the household. Continuing with education requires financial expenditure from the household who are less likely than her own parents to value the importance of her education. Instead, in-laws expressed a wish that young mothers found employment, created their own small businesses or undertook childcare and household chores so that other members of the household could work. In settings where financial stability is uncommon and disposable income does not exist, it is difficult for households to invest in education as a means for future earnings.

Young women in Mozambique fall at the bottom of gender and social hierarchies [7, 25]. Pregnancy in adolescence, specifically among those unmarried, goes against social norms in Mozambique which uphold the importance of being a “good girl” who is obedient, puts her family’s and community’s needs before her own, and sexually chaste [26]. As a result, adolescents and young women who become pregnant face high levels of community stigma and discrimination [25] which increases their risk for negative psychosocial, physical, educational and economic outcomes. Hutchinson [22] and colleagues found young women believed it impossible to improve their wellbeing in the context of an unplanned pregnancy. In our study, poor understanding of maternal and mental health was identified in all stakeholder groups as a challenge to positive perinatal outcomes.

The importance of social support during the perinatal period was viewed as key to positive outcomes by all participants. A South African study of the experiences of adolescent mothers found girls found value in the friendships they developed with other young mothers through the ability to share experiences and support one another [19]. In the United States, greater social support was associated with reduced depression symptoms among adolescent mothers during the year after birth [27]. Lack of parental support was identified as a risk factor for adolescent pregnancy in systematic review of the literature from sub-Saharan Africa [28]. Although most perinatal interventions are delivered individually, group sessions or interactions to encourage friendships between young women and interventions for family members to increase social support may be useful in preventing poor mental health.

Unmet needs in care were identified largely by the service providers interviewed. The majority identified a lack of adolescent-centred and youth friendly services. Due to high levels of stigma associated with early pregnancy, the need for non-judgemental and attractive services to increase antenatal appointment attendance was identified. This perspective is aligned with research from both low and high resource countries [10, 29]. Research has identified young people as having distinct needs and challenges to maintaining good health which are not addressed through traditional services [30]. As a result, youth friendly services have been identified as key to improving adolescent physical and mental health outcomes.

In addition to increased knowledge about the physical and emotional changes that can occur during the perinatal period and how to take care of themselves, participants also identified the need for support in creating positive relationships and problem-solving. Exclusion from education as a result of pregnancy and existing policies create a barrier to developing and strengthening communication skills. This highlights the need for an intervention which extends beyond physical health and parenting to address social determinants of mental health such as education and financial position [31].

Anastas’ [32] review of the narratives of 41 qualitative studies on adolescent pregnancy found the majority of studies prescribed to the view of the girl as the problem. Interventions to curb and address teenage pregnancy stemming from this typology focus on individual change. With respect to adolescent perinatal mental health, interventions aim to improve mental health literacy and provide treatment for disorders. Fewer studies perceived the problem to be rooted in society. Interventions to address this perception of the problem focus on societal and social reforms to improve access to education and employment, reduce gender-based violence and reduce stigma associated with early pregnancy. In our study, stakeholders aligned with both narratives. The need for greater understanding of maternal and mental health, child development and parenting techniques supported the view of the girl as the problem. Conversely, challenges surrounding education, employment and social support are rooted in an understanding of structural barriers and cultural norms which inhibit the positive development of young mothers.

Our co-designed intervention, the Mama Felíz programme, functions as a motherhood preparation course which aims to improve young women’s agency to improve their health and emotional wellbeing, strengthen their communication and problem-solving skills, and build support networks within their families and with other mothers in their communities. It addresses social determinants of mental health (e.g. social support, trauma, and distress) through a strengths-based approach aligned with the Positive Youth Development Framework [33]. The intervention is delivered through a mixture of individual, group and family sessions by local mothers reflects the needs and preferences of young women and their families.

While structural and cultural issues are only partially addressed through additional intervention at the family-level, our future intervention development work will expand from a human-centred design to include a systems-thinking approach. This will address additional social determinants of mental health (e.g. gender, age and social hierarchies) which impact young women’s health, educational, economic, and social outcomes. These interventions will work to enhance the effect of the individual-focused component.

Feasibility and sustainability of the intervention are important components of development and must be addressed to overcome the barriers to scale encountered by preceding mental health interventions. Through close collaboration with service providers and the Ministry of Health, Mama Felíz has been designed to align with and complement existing government initiatives and maternal health services within the district. Although funding has yet to be identified, we hope to procure initial support for implementation and evaluation through grant funding and in-kind support of local stakeholders, including relevant not-for profit organisations.Work to further develop the intervention will continue to rely on close partnership with local stakeholders to identify ways ensure sustainability and scale.

## Conclusions

Mental health interventions are traditionally developed by clinicians and researchers with minimal input and buy-in from those who would receive or provide them. Although a number of interventions have shown promise in small-scale delivery and research, very few interventions have found success in large-scale implementation. The lack of stakeholder involvement may be one of the most important factors contributing to this result. Through an active partnership with young women, their partners and family members and health care representatives we have designed an intervention which is aligned with their needs and priorities and has high stakeholder buy-in.The project represents a relatively new trend in the use of human-centred design within the field of global health and one of the first projects focused on improving mental health in LMIC. It has demonstrated the feasibility of the approach in global mental health and has provided the foundation for further development and testing.

## Data Availability

Data used in analysis can be accessed by request. Requests for data should be sent to the corresponding author (TTS).

## Abbreviations

LMIC: Low and middle-income countries
FGD: Focus group discussion
